# Ketamine effects on resting state functional brain connectivity in major depressive disorder patients: a hypothesis-driven analysis based on a network model of depression

**DOI:** 10.3389/fnins.2025.1531375

**Published:** 2025-02-03

**Authors:** Kasper Recourt, Joop Van Gerven, Nadieh Drenth, Jeroen van der Grond, Kantaro Nishigori, Nic J. Van Der Wee, Gabriël E. Jacobs

**Affiliations:** ^1^Department of Psychiatry, Centre for Human Drug Research, Leiden, Netherlands; ^2^Department of Psychiatry, Leiden University Medical Center, Leiden, Netherlands; ^3^Department of Radiology, Leiden University Medical Center, Leiden, Netherlands; ^4^Sumitomo Pharma Co., Ltd., Tokyo, Japan

**Keywords:** hypothesis-driven analysis, major depressive disorder, ketamine, functional MRI, glutamatergic modulator, NMDA receptor

## Abstract

**Introduction:**

Ketamine demonstrates robust and rapidly occurring antidepressant effects in patients with difficult-to-treat major depressive disorder. Ketamine’s antidepressant effects and its impact on functional networks in non-resistant forms of major depressive disorder are expected to provide valuable insight into ketamine’s mechanism of action related to depression.

**Methods:**

This study employs an existing network model of major depressive disorder to investigate the effects of ketamine on resting state connectivity in a therapy-non-resistant major depressive disorder population. In a randomized, double-blind, placebo-controlled, cross-over study, 0.5 mg/kg racemic ketamine or 0.9%NaCl was administered intravenously in 16 MDD patients. We applied resting-state functional magnetic resonance imaging (rs-fMRI) to explore changes in functional brain connectivity directly at 50, 80 and 165 min (acute) and 24 h (delayed) following ketamine administration. A clinician-rated 10-item scale (MADRS) was administered at 165 min and 24 h after ketamine administration. Connections-of-interest (COIs) were based on the previously published corticolimbic-insular-striatalpallidal-thalamic (CLIPST) circuitry model of major depressive disorder.

**Results:**

Compared with placebo, ketamine significantly (*p* < 0.0014) reduced the mean (SD) MADRS total score from 21.2 (5.9) pre-dose to 10.3 (4.6) 24 h post-dose. At both acute (*p* < 0.0172) and delayed (*p* < 0.0488) time points, significant rs-fMRI connectivity changes occurred only in MDD-related COIs as proposed by the CLIPST model. No changes in functional connectivity were found in non-CLIPST connections.

**Discussion:**

This study demonstrates that ketamine specifically affects depression-related circuitry. Analyzing functional connectivity based on a neurocircuitry model of a specific CNS disease and drug action may be an effective approach that could result in a more targeted analysis in future pharmaco-fMRI studies in CNS drug development.

## Introduction

The non-competitive glutamatergic N-methyl-D-aspartate receptor (NMDAR) antagonist ketamine is typically available as a racemate of the enantiomers R-(−)-ketamine and S-(+)-ketamine (Stahl, 2013). Since both racemic ketamine and enantiomer S-(+)-ketamine demonstrate rapidly occurring and robust mood improvement unrelated to euphoria (Zarate et al., 2006; Wilkinson and Sanacora, 2019; Bahji et al., 2021), these compounds present fast-acting pharmacological alternatives to monoaminergic treatment options for depression (Trivedi et al., 2004; Feyissa et al., 2009; MacHado-Vieira et al., 2010; Lapidus et al., 2013). In fact, S-(+)-ketamine has recently been approved by the American Food and Drug Administration (FDA; Kim et al., 2019) and the European Medicines Agency (EMA; Mahase, 2019) for difficult-to-treat depression. Nevertheless, although ketamine can arguably be regarded as a prototype for an entirely new class of antidepressant medications, how it affects functional brain connectivity changes in regions that are implicated in the pathophysiology of depression is still a matter of debate (Krystal et al., 2019). By distinguishing the mechanistic basis for ketamine’s acute, undesirable effects from its more sustained antidepressant activity, its ultimate therapeutic effects related to functional networks in major depressive disorder (MDD) may be elucidated.

**Figure 1:**
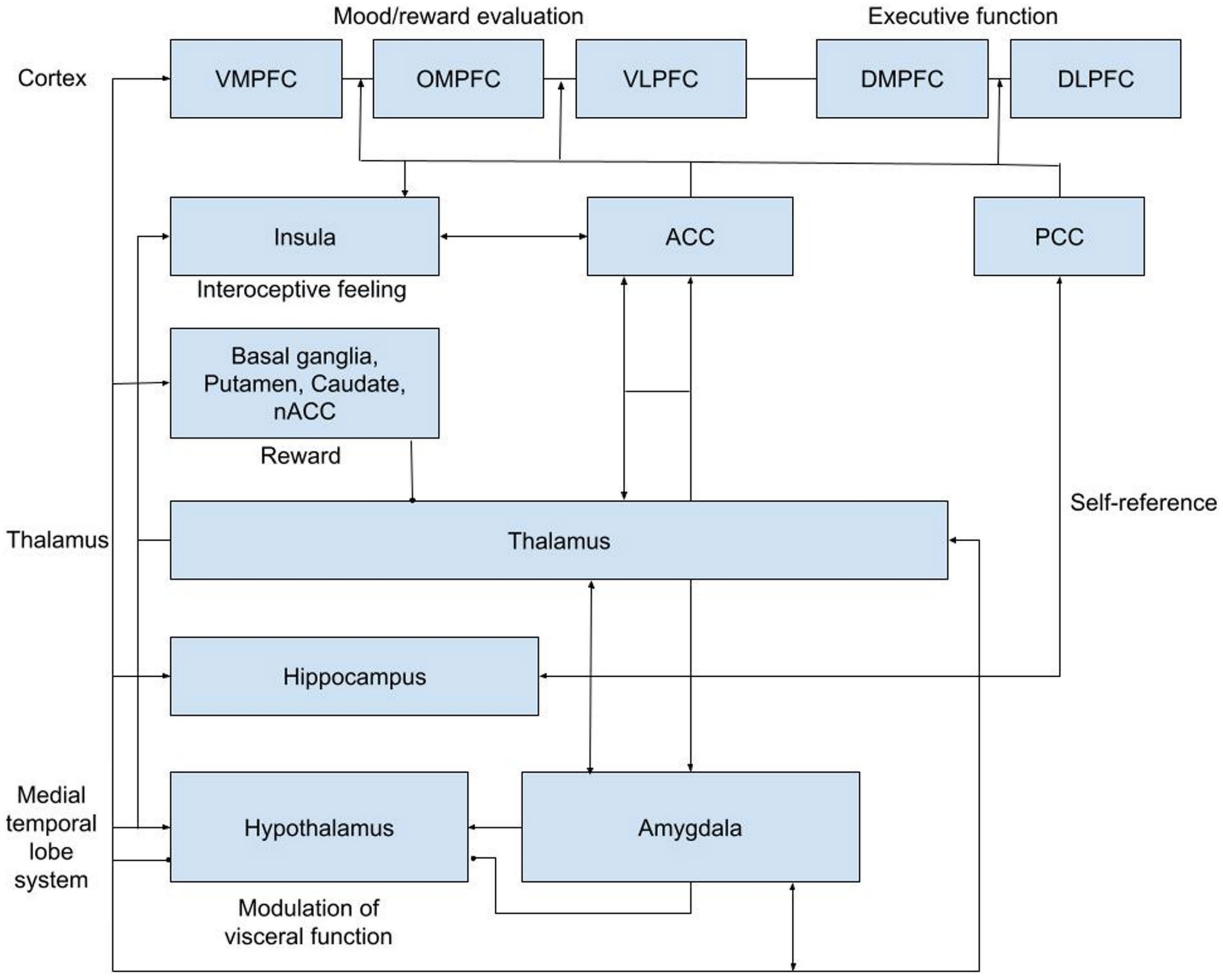
CLIPST network hypothesis. ACC, anterior cingulate cortex; DLPFC, dorsal lateral prefrontral cortex; DMPFC, dorsal medial prefrontral cortex; NAcc, nucleus accumbens; OMPFC, orbitomedial prefrontral cortex; PCC, posterior cingulate cortex; VLPFC, ventral lateral; prefrontral cortex; VMPFC, ventral medial prefrontral cortex.

Both acute and delayed effects of ketamine on neural responses have been studied using PET and fMRI in MDD patients and healthy volunteers, showing enhanced responses to positive emotions (Kraus et al., 2020; Abdallah et al., 2021, 2022; Murrough et al., 2015). Decreased functional connectivity within the default mode network (DMN) to key regions like the dorsal nexus, pregenual anterior cingulate cortices, medial prefrontal cortex and posterior cingulate cortices was observed, indicating neuronal normalization in MDD (Sundermann et al., 2014; Scheidegger et al., 2016). This pattern resembles changes seen after a single SSRI dose (Klaassens et al., 2015). While ketamine typically induces concentration-related psychomimetic and dissociative effects after a single acute intravenous administration (Kleinloog et al., 2015), its antidepressant effects peak within 24 h and persist for up to 10 and 21 days on average (Zarate et al., 2006). Because of this pattern, altered connectivity may underlie its role in reducing depressive symptoms and represent a systems-level treatment mechanism (Scheidegger et al., 2016) Since these antidepressant effects persist even after ketamine and its active metabolites have been eliminated from the central nervous system (CNS), we formed the hypothesis that initial NMDAR-mediated changes induce alterations in brain connectivity that play a critical role in reducing depressive symptomatology. The focus of several important neurocircuitry-based models for depressive disorders put forward reciprocal interactions between ventral and the highly integrated dorsal circuits of the prefrontal cortex (PFC) and their respective interactions with elements of the limbic system, basal ganglia, insula, and hypothalamic–pituitary–adrenal (HPA) axis (Bench et al., 1992; Drevets, 2000; Davidson et al., 2002; Mayberg, 2002, 2007; Anand et al., 2005; Drevets et al., 2008; Koenigs and Grafman, 2009). Of particular interest is the neurocircuitry-based corticolimbic-insular-striatalpallidal-thalamic circuitry (CLIPST) model (Figure 1; Vago et al., 2011), which relates functional-anatomical neurocircuits that have been consistently implicated in the (patho)physiology of mood regulation and depression. The CLIPST model is composed of closely defined and interlinked functional-anatomical components that could be related and easily translated to a resting state functional magnetic resonance imaging (rsfMRI)-network analysis. In this context, MDD can be conceptualized as an imbalance in one or more of these interacting regions, where specific symptoms or clusters of functionally related symptoms can (theoretically) be linked to abnormalities in matching network components. Therefore, changes in functional brain connectivity following ketamine administration within the CLIPST model could potentially reflect NMDAR-mediated network connectivity changes related to ketamine’s antidepressant effects. This led to a relatively simple but robust approach to perform a more targeted analysis of pharmaco-rs-fMRI data.

Importantly, the antidepressant effects of ketamine have been predominantly investigated in patients with therapy-resistant forms of both unipolar MDD and bipolar depression (Zarate et al., 2006, 2012; Diazgranados et al., 2010; Valentine et al., 2011; Mathew et al., 2012; Murrough et al., 2013a, 2013b; Rasmussen et al., 2013; Permoda-Osip et al., 2014). As such, these populations represent a relatively specific subgroup of MDD that has been extensively treated with monoamine modulators, lithium and/or electroconvulsive therapy over a period of many years (Fava and Davidson, 1996). Therefore, demonstration of ketamine’s antidepressant effects and its impact on functional networks in non-resistant forms of MDD is expected to provide valuable insight into ketamine’s mechanism of action related to depression in a broader sense.

In summary, the current study in MDD patients was designed to identify the effects of racemic ketamine over time, which are likely to be related to specific underlying pathophysiological network alterations. We hypothesized that, given the robust and sustained antidepressant effects of ketamine, potential network alterations would be more pronounced in networks shown to be associated with MDD compared with other networks not previously implicated in MDD. For this hypothesis-based analysis, the previously described CLIPST network model of depression was selected to define the functional network outcomes for the current study (Vago et al., 2011). The CLIPST model is composed of closely defined and interlinked functional-anatomical components that could be related and easily translated to a resting state functional magnetic resonance imaging (rsfMRI)-network analysis. This led to a relatively simple but robust approach to perform a more targeted analysis of pharmaco-rs-fMRI data.

## Materials and methods

### Study design

**Figure 2:**
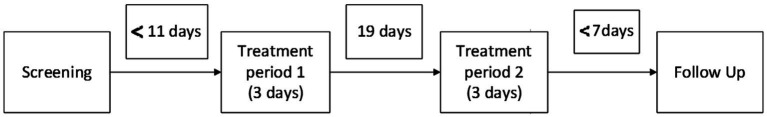
Study design.

This was a randomized, double-blind, placebo-controlled, 2-period, 2-treatment cross-over study. Ethical and regulatory approval was obtained from the Stichting Beoordeling Ethiek Biomedisch Onderzoek (BEBO, Assen, the Netherlands) and the Central Committee on Research involving Human Subjects (CCMO, Den Haag, the Netherlands). The study was registered at the Dutch competent authority (CCMO) trial register^1^ under eudraCT number 2016–003999-51. Informed consent was obtained from all patients. The total duration of the study for each patient was a maximum of 42 days and consisted of a screening examination (day −11 to −1) at baseline, two treatment periods of 3 days with a washout period of 21 days between study drug administrations (19 days between treatment periods) to prevent carry-over effects, and a follow up after a minimum of 4 days and a maximum of 7 days following the last treatment period. Patients were interviewed by telephone once a week between the two treatment periods. The randomization code was generated using SAS version 9.4 by a study-independent statistician and was kept strictly confidential. The study design is illustrated in Figure 2.

### Participants

Medicated male and female outpatients, aged 18 to 65 years, diagnosed with major depressive disorder (MDD), who demonstrated partial or non-response to a first trial with an SSRI or SNRI despite a therapeutic dose for at least 4 weeks of treatment, were included. Patients were required to be in acceptable physical health as determined at screening by medical history, physical examination, blood laboratory results and electrocardiogram. Patients met the Diagnostic and Statistical Manual of Mental Disorders, Fourth Edition (DSM-IV) diagnostic criteria for MDD, without psychotic features based upon clinical assessment at screening and confirmed by their treating psychiatrist and/or general practitioner and by the Mini-International Neuropsychiatric Interview (MINI).(Sheenhan et al., 1998), with a Hamilton Rating Scale for Depression (HAM-D) severity score of ≥18, at screening and pre-dose on both treatment periods. Comorbid anxiety disorders, except for post-traumatic stress disorder (PTSD), were allowed, provided these were secondary diagnoses to MDD. Patients were excluded if they had undergone involuntary psychiatric hospitalization during the current episode, were diagnosed with bipolar or related disorders, obsessive-compulsive disorder, intellectual disability, borderline personality disorder, antisocial personality disorder, histrionic personality disorder or narcissistic personality disorder, or had a history of drug or alcohol abuse or dependence. Patients were tested for drugs of abuse at screening and prior to treatment (pre-dose). Contraindications for MRI scans or any cerebral and/or head abnormalities were exclusionary.

### Study drugs

Racemic ketamine (0.5 mg/kg) or 0.9% sodium chloride as matching placebo was administered intravenously over 40 min on either of the two study periods. Patients received two intravenous lines (in separate arms); one for drug infusion and one for blood sampling. A Graseby™ 3200 syringe pump was used to administer 40 ml of ketamine or saline intravenously at a rate of 1 ml/min. Any other medication taken during the study was reported to the investigator. No new antidepressant therapy was started between screening and the follow-up visit, and the dose of current antidepressant therapies had to remain unchanged for the duration of the study. No prescription medication other than allowed antidepressants (SSRI or SNRI) was permitted either within 14 days prior to study drug administration or less than 5 half-lives of the prescribed drug and throughout the study.

### Pharmacodynamic assessments

The Montgomery-Asberg Depression Rating Scale (MADRS; Montgomery and Åsberg, 1979) was the primary efficacy evaluation. The MADRS is a clinician-rated scale designed to measure depression severity and detect changes due to antidepressant treatment. The scale consists of 10 items, each scored from 0 (item not present or normal) to 6 (severe or continuous presence of the symptoms), for a total score of 60. The MADRS was administered at screening, during both visits at baseline, at 165 min and 24 h after ketamine administration, during the weekly telephone interviews between treatment periods and at follow-up. All staff involved in the study were trained in using the Structured Interview Guide for the MADRS (SIGMA) to ensure consistency and reliability in administering the Montgomery-Asberg Depression Rating Scale (MADRS). This standardized approach enhanced the validity of depression severity assessments throughout the study.

The Clinician-Administered Dissociative States Scale (CADSS; Bremner et al., 1998) is a 28-item scale for assessing dissociative states. The CADDS was used as a change measure to assess dissociative states before and after treatment. The CADSS was administered during screening and both visits at baseline, at 165 m and 24 h, during the weekly telephone interviews between treatment periods and at Bond and Lader VAS, as originally described by Norris, have been used to quantify subjective effects of a variety of sedative agents (De Visser et al., 2001). The Bowdle VAS (Bowdle et al., 1998) has been used to study glutamatergic drug effects. These measurements calculated three main factors: internal perception, external perception and feeling high (Kleinloog et al., 2015). Both the Bowdle VAS and Bond and Lader VAS were administered electronically at screening, baseline, pre-dose, at 40,65,96,150 and 240 min and 24 h after ketamine administration.

Resting-state functional Magnetic Resonance Imaging (fMRI) scans were acquired at the Pharmacological MRI Unit of the Leiden University Medical Center (LUMC). Prior to the first fMRI session, patients found eligible for the study underwent a mock scan at the dummy scanner (a decommissioned MRI scanner without magnetic field). Actual scans were acquired on a 3-Tesla Philips Ingenia Scanner (Philips Medical System, Best, the Netherlands) equipped with a 32-channel head coil. Resting-state functional images were acquired at each visit on fixed time points: one pre-dose scan (baseline) and four post-dose scans at 50 min, 80 min, 165 min, and 24 h. To avoid between-scan variations in field-of-view (FOV), a smart scan protocol (available on Philips Ingenia scanners) was used, which repositions the FOV based on the first scan of the subject. Participants were instructed to lie as still as possible, close their eyes and not to fall asleep while T2*-weighted whole-brain volumes were obtained using a single-shot echo-planar sequence with repetition time (TR) set to shortest (varying between 2,289 and 2,398 milliseconds [ms]), echo time (TE) = 30 ms, flip angle = 85°, 40 slices, FOV 220 × 200 × 137 millimeter [mm], voxel size 3.44 × 3.44 × 3.13 mm, 220 volumes, and a scan duration of 8 min and 55 s. In addition, a high-resolution three-dimensional anatomical image was obtained at baseline for structural reference, with TR = 9.8 ms, TE = 4.6 ms, flip angle = 8°, 140 slices, FOV = 224 × 179 × 168, voxel size 0.88 × 0.88 × 1.20 mm, and a scan duration of 4 min and 58 s.

### Pharmacokinetic assessments

Pharmacokinetic (PK) samples were taken at −5, 20, 30, 40, 50, 65, 95, 240, and 1,440 min from ketamine administration during both study periods. Isolation of ketamine, norketamine, hydroxynorketamine (HNK), and reference compounds ketamine-d4 and norketamine-d4 from human K2-ethylene diamine tetra-acetic acid (EDTA) plasma was performed by protein precipitation. The purified sample was analyzed by Ardena Bioanalytical Laboratory (ABL) Assen, the Netherlands, using an API 6500+ LC–MS/MS system.

### Safety

Treatment-emergent adverse events (TEAEs) were monitored with special attention for suicidal ideation using the C-SSRS (Posner et al., 2007). Physical examination, body weight, supine vital signs, digital pulse oximetry, 12-lead electrocardiogram (ECG) and continuous ECG monitoring (during study drug administration) were performed. Blood samples for serum chemistry and hematology and a urine sample for urinalysis were collected at baseline and set time points throughout the study.

### Statistical analysis

Since this was an exploratory study, sample size was not based on a formal power calculation. However, based on previous CHDR studies, we performed CHDR with ketamine (Niesters et al., 2012; Kleinloog et al., 2015)^.^ A sample size 16 was considered adequate to show pharmacodynamic and rs-fMRI effects. All safety, PK and efficacy statistical programming, except for the fMRI analysis, was conducted with SAS 9.4 for Windows (SAS Institute Inc., Cary, NC, USA). Non-compartmental PK analysis was performed with R2.12.0 for Windows (R Foundation for Statistical Computing, Vienna, Austria; R Development Core Team, 2010).

All repeatedly measured Pharmacodynamic (PD) endpoints (VAS and questionnaires) were summarised by treatment and time. PD parameters were initially analyzed without transformation, but log transformation was applied if the data suggested otherwise. Each parameter was analyzed with a mixed model analysis of covariance (ANCOVA) with treatment, time, period and treatment by time as fixed factors and subject, subject by treatment and subject by time as random factors and the (average) baseline measurement as covariate. The Kenward-Roger approximation was used to estimate denominator degrees of freedom, and model parameters were estimated using the restricted maximum likelihood method. The general treatment effect and specific contrasts were reported with the estimated difference and the 95% confidence interval, the least square mean estimates and the *p*-value. The contrast ketamine–placebo was calculated within the model.

How the fMRI data were preprocessed and analyzed can be found in the Supplementary material. We performed a hypothesis-driven analysis restricted to changes in connectivity between functionally connected areas based on a predefined network model of MDD according to the existing CLIPST model (Vago et al., 2011). To investigate region-to-region connectivity for connections of interest (39 total, see Table 1), masks of all regions together with a CSF and WM mask for nuisance reduction were entered into a dual regression. In the dual regression, all masks were regressed into each subject’s preprocessed rs-fMRI scan, resulting in subject-specific time series for each region. These time courses were used to calculate partial correlations (Fisher’s *r*-to-*z* transformed) between the regions at each time point as measures of connectivity using the FSLNets package.^2^ The partial correlations (*z* scores) of the connections of interest were extracted from FSLNets and transferred to SPSS (version 25.0) for further analysis. Next, paired t-tests were performed for each connection of interest at the acute (results from 5, 80, and 165 min were pooled for this purpose) and the delayed (at 24 h) time points by contrasting the baseline corrected *z* score of the ketamine condition versus the baseline corrected *z* score of the placebo condition. The connections of interest were chosen from a ‘grid’ of 15×15 possible connections between all regions analyzed in this study (Table 1). The 39 connections of interest were identified based on the CLIPST network hypothesis (Figure 1).

**Table 1:** Connections of interest (indicated by numbers 1–39) in a 15×15 (105 connection) grid.

	dmPFC	dlPFC	omPFC	vmPFC	vlPFC	Hypothalamus	ACC	PCC	Insula	Amygdala	Hippocampus	Thalamus	Nacc	Putamen	Caudate
dmPFC		1	2	3	4			5							
dlPFC			6	7	8		9								
omPFC				10	11	12	13	14	15		16	17	18		
vmPFC					19	20	21	22	23		24	25	26		
vlPFC						27	28		29		30	31	32		
Hypothalamus									33	34					
ACC									35	36	37	38			
PCC															
Insula															
Amygdala												39			
Hippocampus															
Thalamus															
Nacc															
Putamen															
Caudate															

Further details of the selection approach can be found in the Supplementary material. We hypothesized that ketamine would cause more significant changes in the functional connectivity between the 39 connections of interest within the CLIPST model than between the regions unrelated to mood, according to the CLIPST model (66 connections). This hypothesis was tested using a Fisher exact test.

The following individual PK parameters were determined based on the concentration versus time curves: the area under the curve (AUC) was computed from 0 to the last measurement point (AUC0-last). The terminal half-life was estimated and AUC zero to infinity (AUC0–inf) was derived from AUC0-last and the extrapolated area from the last measurement point to infinity based on the terminal half-life. Additionally, maximum concentration (Cmax) and time of maximum concentration (Tmax) were determined. PK parameters were summarised by treatment group, with the number of observations, mean, SD, median, Min, and Max for each time point of measurement. Individual racemic plasma ketamine, norketamine and hydroxynorketamine (HNK) concentrations were plotted versus time per individual using both a linear and log y-axis. Additionally, concentration versus time curves were plotted per treatment group with the group median added.

All reported adverse events (AEs) with onset during the treatment were included in the analysis. For each AE, the percentage of subjects who experienced at least one occurrence of the given event was summarised by treatment group and summary statistics were provided. Vital signs, ECG and clinical laboratory test values that were out of range and considered clinically significant were reported as adverse events.

## Results

### Participants

**Figure 3:**
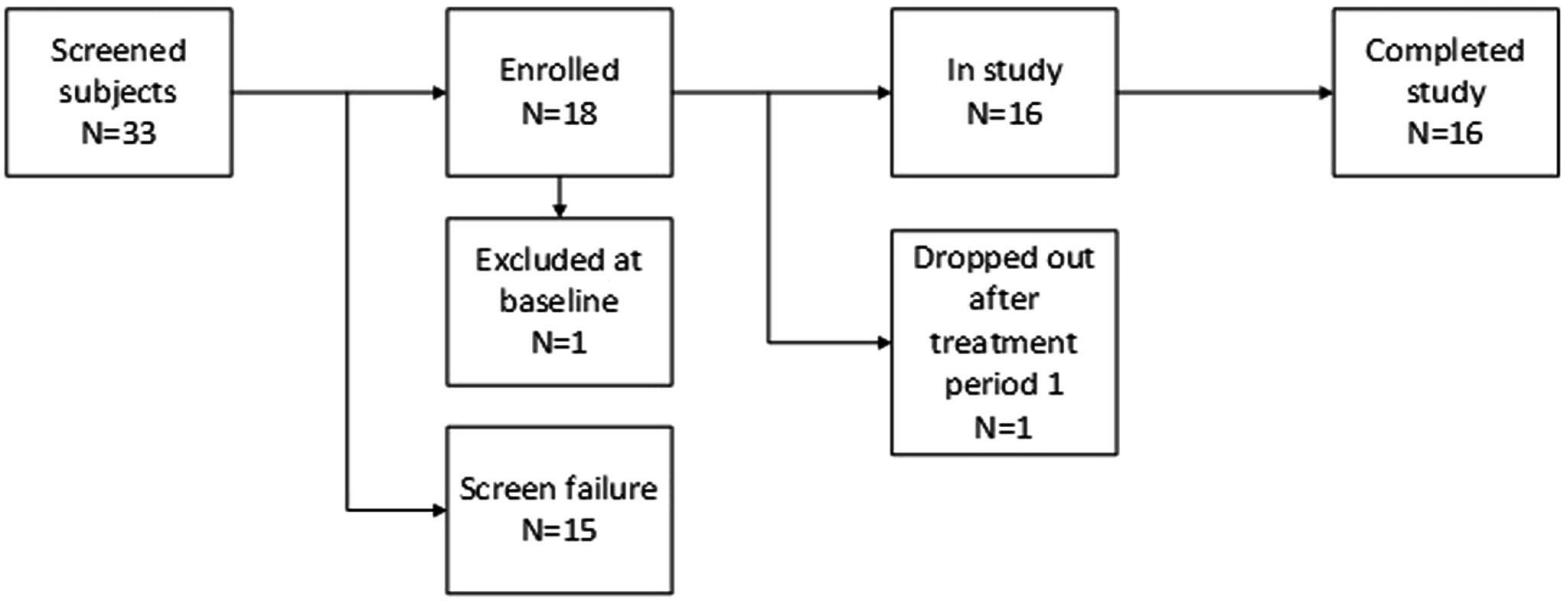
Disposition schedule.

Thirty-three patients were assessed for eligibility. Fifteen patients were excluded based on the in- and exclusion criteria. One patient was found ineligible at baseline due to a positive urine drug test for opiates, and a second patient withdrew consent after the first visit due to worsening depressive symptoms. Of the eligible patients, a total of 16 patients completed the study. The disposition schedule for the study can be found in Figure 3.

In total 8 (47%) male and 9 (53%) female patients, aged mean (SD) 26.8 (7.3) and with a BMI of mean (SD) 23.5 (3.3), were enrolled in the study and received at least one dose of study treatment. All patients were being treated with SSRIs, and none with SNRIs. The prescribed SSRIs (% of patients, average doses) were sertraline (35%, 100 mg), citalopram (29%, 22 mg), escitalopram (18%, 20 mg), fluoxetine (12%, 20 mg) and paroxetine (6%, 20 mg). The average duration of the current depressive episode was 11 months. Patients were included based on depression severity using the HAM-D17 total score, and the MADRS total score was the main efficacy measure. The mean (SD) MADRS score was 28.1 (3.1), 21.9 (6.6), and 19.0 (5.0) at screening and baseline of the first and second study periods, and the mean (SD) HAM-D17 score was 20.2 (2.5) and 19.9 (2.0) at screening and day 1 of the first study period, respectively.

### Pharmacodynamic outcomes

**Figure 4:**
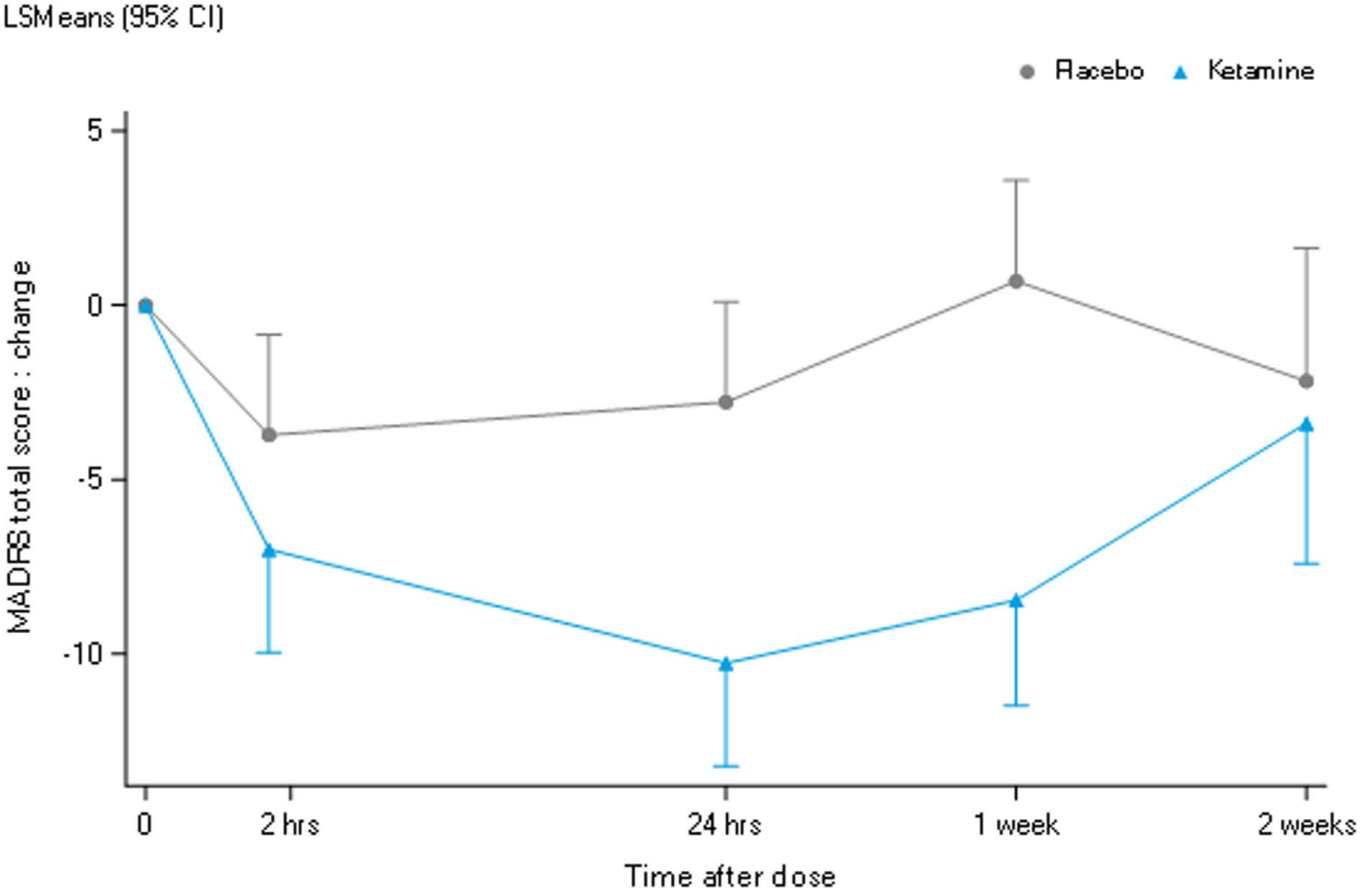
LSM change from baseline MADRS total score.

As shown in Figure 4, compared to placebo, the mean baseline-corrected MADRS total score was significantly reduced at 24 h and 1 week but not 2 weeks, and over the total two-week period following ketamine administration. The responder criterion of a decrease in the MADRS total score compared to baseline of ≥50% was met by 56% (9/16), 44% (7/16), and 25% (2/8) of patients at 24 h, 1 week and 2 weeks after ketamine administration, respectively.

**Figure 5:**
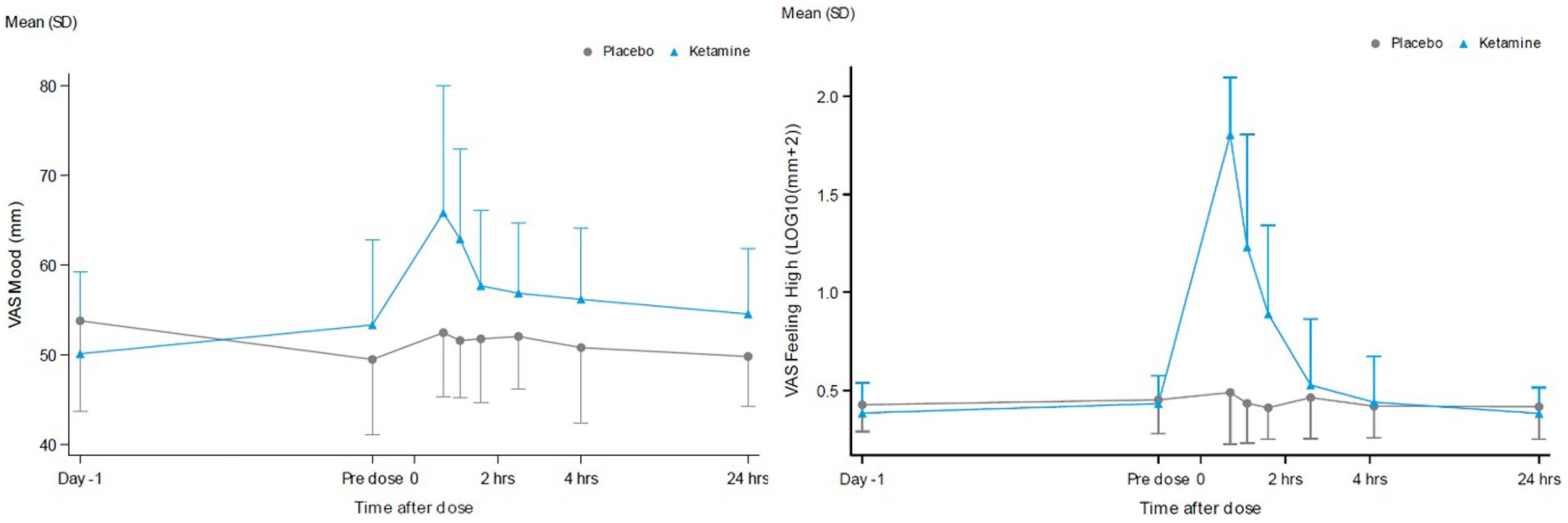
VAS scores for the contrast ketamine versus placebo over 24 hours for VAS mood **(A)** and VAS feeling High **(B)**.

Dissociative effects, as measured by the CADSS, were observed to peak following administration at the 165-min assessment and returned to baseline at 24 h after ketamine administration, while placebo did not affect the CADSS. This timing reflects when the retrospective assessment of dissociative experiences was conducted, rather than a strict two-hour post-infusion peak. VAS alertness, VAS internal perception, VAS external perception, VAS feeling high and VAS Mood peaked around 60 min after ketamine administration and returned to baseline after 2 h. VAS Mood and the most pronounced psychomimetic effect, VAS feeling high, are shown in Figure 5. Compared with placebo, ketamine increased VAS mood, VAS internal perception, VAS external perception and VAS feeling high statistically significantly (Table 2).

**Table 2:** VAS scores for the contrast ketamine versus placebo over 24 h: estimated Least Square Means (LSMs), estimated difference with 95% confidence.

Parameter	Least Square Means (LSM)		Estimated difference with 95% Confidence interval	Significance
	placebo	ketamine		
VAS alertness (mm)	47.55	46.82	−0.73 (−5.89, 4.42)	*p* = 0.7575
VAS mood (mm)	51.34	58.90	7.55 (3.55, 11.56)	*p* = 0.0018
VAS calmness (mm)	55.86	60.31	4.45 (−1.04, 9.94)	*p* = 0.1030
VAS internal perception [LOG10 (mm + 2)]	0.43	0.54	0.11 (0.012, 0.20)	*p* = 0.0294
VAS external perception [LOG10 (mm + 2)]	0.40	0.62	0.23 (0.11, 0.34)	*p* = 0.0007
VAS feeling high [LOG10 (mm + 2)]	0.43	0,89	0.45 (0.29, 0.61)	*p* < 0.0001

At the pooled acute timepoint, statistically significant differences in functional connectivity for ketamine compared with placebo were found for the pairs (Figure 1): vmPFC and NAcc [t(15) = 3.193, *p* = 0.006], omPFC and thalamus [t(15) = −2.588, *p* = 0.021], vlPFC and thalamus [t(15) = −3.325, *p* = 0.005], and omPFC and dlPFC [t(15) = −2.408, *p* = 0.029]. Furthermore, the connectivity between vmPFC and NAcc was positive for ketamine (M = 1.12, SD = 1.46) and negative for placebo (M = −0.67, SD = 1.81). Negative connectivity for ketamine versus positive connectivity for placebo was found for the omPFC and thalamus (ketamine M = -1.35, SD = 1.82; placebo M = 0.03, SD = 1.82), vlPFC and thalamus (ketamine M = -1.26, SD = 2.03; placebo M = 0.44, SD = 1.38), and omPFC and dlPFC (ketamine M = -0.42, SD = 1.99; placebo M = 0.91, SD = 1.39).

At the delayed timepoint, statistically significant differences in functional connectivity for ketamine compared with placebo were found for the pairs: omPFC and PCC [t(15) = 2.648, *p* = 0.018], omPFC and vmPFC [t(15) = −2.257, *p* = 0.039], and ACC and insula [t(15) = −2.392, *p* = 0.030]. The correlation between omPFC and PCC was positive for ketamine (M = 1.40, SD = 1.91) and negative for placebo (M = -0.41, SD = 2.07). Negative connectivity for ketamine versus positive connectivity for placebo was found for the omPFC and vmPFC (ketamine M = -0.86, SD = 2.34; placebo M = 1.21, SD = 2.97), and ACC and insula (ketamine M = -0.26, SD = 2.36; placebo M = 1.20, SD = 2.04).

At the acute time point, there were 4 significant effects within the connections of interest (39) and 0 outside the connections of interest (66; *p* = 0.0172). At the delayed time point, there were 3 significant effects within the connections of interest and 0 outside the connections of interest (*p* = 0.0488).

### Pharmacokinetic outcomes

**Figure 6:**
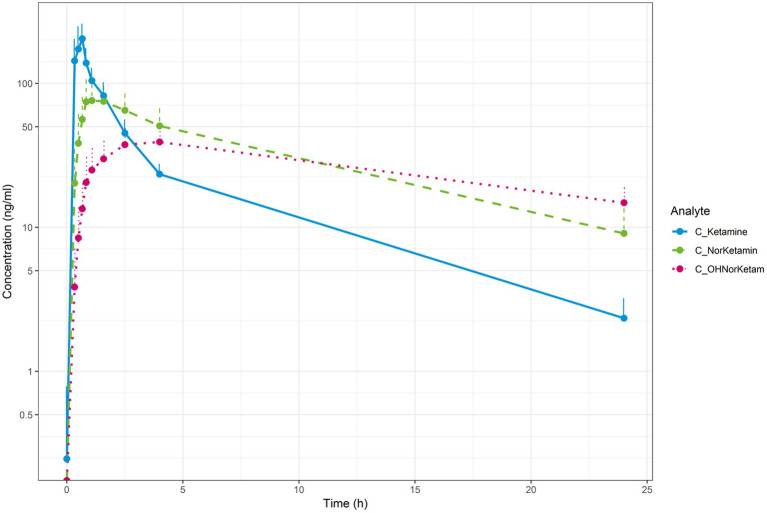
Mean concentration-time profiles for plasma ketamine (blue), plasma norketamine (green) and plasma (OH)-norketamine (pink; semi-log).

The mean concentration-time profiles for plasma ketamine, norketamine and HNK are shown in Figure 6. Mean (SD) plasma ketamine, norketamine and HNK reached a mean Cmax of 208.56 (54.85), 84.96 (24.02), and 39.58 (10.46) ng/ml, at 0.64, 1.39, and 3.46 h, respectively. The mean apparent terminal half-life for plasma ketamine and norketamine was 5.26 and 7.51 h, respectively. Plasma ketamine had the highest apparent clearance, followed by norketamine, while apparent clearance could not be determined for plasma HNK. In general, Tmax and apparent terminal half-life were more variable for plasma norketamine and HNK compared to ketamine. The area under the plasma concentration-time curve (AUC) mean (SD) from time of dosing to the last observation, calculated using the linear trapezoidal method and the terminal half-life was 557 (84) ng*h/ml for ketamine, 824 (248) ng*h/ml for norketamine and 643.55 (144.33) ng*h/ml for HNK.

### Safety outcomes

Overall, no clinically relevant changes or unexpected treatment-related trends were observed following ketamine administration. There were no clinically significant changes from pre-dose values in any of the vital signs, ECG recordings, C-SSRS assessments, clinical chemistry, hematology or urinalysis safety assessments following administration of ketamine or placebo. A total number of 91 TEAEs were reported; 86 were in the ketamine treatment group, and 5 were in the placebo treatment group. No serious adverse events occurred, with the most frequently occurring TEAEs judged to have a relationship to ketamine being dissociation (68.8%) and dizziness (50.0%), all of which were self-limiting and of mild severity. Paracetamol was used in several cases to treat headaches, and no other concomitant medication was used to treat AEs.

## Discussion

The present study employed an existing network model of depression to investigate the resting state fMRI effects of a single ketamine administration in a stably medicated, moderate to severe MDD outpatient population that demonstrated partial or non-response to a first trial with an SSRI or SNRI. The study was a proof of concept designed to test various measurement and analysis techniques and to gain a better understanding of the effect of ketamine with a limited number of participants and measurements. To obtain robust results from this limited study design, with many different, repeated measurements within a small sample size, we used an analysis method that looked at whether ketamine has a more significant effect on the functional connectivity between brain regions involved in MDD as described in the CLIPST model, than on random connections between these brain regions. The method, based on a model without quantitative evidence and whose selection of connections is not validated and may not contain all relevant connections, is a simplified and pragmatic way to measure the effect of a compound in the brain on a disease. This simple dichotomous comparison provides a powerful method to determine whether a compound affects connections involved in a disease more than random other connections. In this study, which sought to test measurement and analysis techniques rather than provide definitive findings, the methodological decisions, including the absence of certain statistical corrections and the choice of analysis techniques, were deliberately tailored to align with the study’s objectives and practical constraints. This approach facilitated a focused investigation of ketamine’s potential effects on specific brain connections, setting the groundwork for future, more detailed analyses.

The hypothesis-driven analysis focused on relevant connections between regions involved in MDD as described by the CLIPST model (Vago et al., 2011) and showed several significant ketamine-induced effects. These significant changes were limited to the 39 MDD-related connections, while no significant changes in functional connectivity were found in the 66 connections outside the CLIPST model. This was significant at both the acute (4/39; *p* < 0.0172) and delayed (3/39, *p* < 0.0488) time points and demonstrates that ketamine specifically affects depression-related circuitry.

Since ketamine previously demonstrated robust concentration-dependent acute psychomimetic and dissociative effects on the Visual Analogue Scales and CADSS in a healthy volunteer study conducted by our group (Kleinloog et al., 2015), these assessments were integrated into the current study to assess such effects in MDD. As expected, based on literature reporting acute adverse effects with ketamine (Acevedo-Diaz et al., 2020) and consistent with the pharmacokinetics of ketamine following intravenous administration over 40 min in MDD, psychomimetic effects peaked at the end of the intravenous infusion. They diminished within 2 h after initiating the infusion. In contrast, however, the time course of ketamine’s antidepressant effects did not follow the pharmacokinetics of either ketamine, norketamine or HNK, as maximal antidepressant effects in the majority of patients were observed 24 h following ketamine administration and persisted up to a week before returning to baseline by 2 weeks. These differential pharmacodynamic effects over time further support the hypothesis that the primary NMDAR-mediated changes induce alterations in brain connectivity that play a critical role in reducing and sustaining depressive symptomatology in MDD.

Plasma ketamine, norketamine and HNK reached a mean Cmax of 208.56, 84.96 and 39.58 ng/ml at 0.64, 1.39 and 3.46 h, respectively. From dosing time to the last observation, the AUC mean was 557 for ketamine, 824 for norketamine and 643 ng*h/ml for HNK. These pharmacokinetic findings were in line with literature (Peltoniemi et al., 2016; Farmer et al., 2020). Furthermore, the responder criterion of a decrease in the MADRS total score compared to baseline of ≥50% was met by 56%(9/16) of patients at 24 h, which is in line with the findings (52%) of a meta-analysis of nine high-quality studies that included 368 patients (Han et al., 2016). Together, in our patient population, the PK of ketamine and its active metabolites and response rate to ketamine were in line with published literature reporting pharmacokinetics and efficacy in relatively more treatment-resistant forms of MDD.

Some limitations of the current study deserve mentioning. Patients were treated with relatively low therapeutic doses of conventional antidepressant drugs, raising the concern whether partial or non-response was not related to undertreatment rather than treatment resistance. Since the response rate to ketamine in the current MDD patient sample was comparable to that reported in literature based on more treatment-resistant forms of MDD, undertreatment seems less likely, as a lower response rate to ketamine would arguably be expected in the case of undertreatment. The decision to include patients with a minimum of 4 weeks of antidepressant treatment, instead of the standard 6 weeks for treatment-resistant depression, aimed to improve recruitment and broaden the findings’ applicability. Unlike prior research focusing on therapy-resistant MDD and bipolar depression, this study targets patients with non-resistant MDD to explore ketamine’s antidepressant effects and its impact on functional networks. This approach seeks to provide insights into ketamine’s broader potential to alleviate depressive symptoms across a wider spectrum of MDD. Furthermore, as the non-response rate was significant in our patient population, pharmacokinetic variability should be considered an important contributing factor. However, in our sample we found no significant difference between responders and non-responders in pharmacokinetic variability with a Cmax mean (SD) coefficient of variation (%CV) of 211 (48) 23 for ketamine, 92 (24) 26 for norketamine and 42 (9) 21 for HNK in responders and a Cmax mean (SD) %CV of 205 (66) 32 for ketamine, 76 (23) 30 for norketamine and 37 (12) 33 for HNK in non-responders. Alternatively, it is not inconceivable that ketamine may affect different brain regions in responders and non-responders and, as a consequence, be more effective in some individuals. Due to the rather limited power in this small sample size, such differential effects were indistinguishable. The small sample size inherently constrains the statistical power and generalizability of the findings. However, this aligned with the exploratory and hypothesis-driven nature of the study. The study did not include a detailed subgroup analysis to differentiate connectivity changes between responders and non-responders to ketamine. While preliminary observations suggest heterogeneity in clinical outcomes, the limited sample size precluded a robust statistical exploration of these differences. Future studies with larger cohorts should aim to stratify participants by response status to identify potential biomarkers or neural correlates of treatment efficacy. Such analyses will provide deeper insights into ketamine’s mechanism of action and may inform more personalized treatment approaches.Despite these limitations, our results were clearly in accordance with findings from earlier studies in predominantly treatment-resistant forms of unipolar MDD and bipolar depression. Such may cause more consideration for future work in non-resistant forms of MDD, given the obvious advantages in terms of recruitment and in addition, the possibility to explore the relationship between symptoms and specific brain regions in larger patient populations and with longer follow-up periods.

The current, relatively small study, analyzing functional connectivity based on an existing neurocircuitry model of CNS disease and drug action, demonstrated disease-specific effects of the non-competitive NMDAR antagonist ketamine that could not be shown with exploratory fMRI connectivity analysis. In principle, such model-based approaches could result in more targeted analyses in pharmaco-MRI studies. Network analysis based on disease connectivity models could potentially also be suitable for preclinical disease models or other neuropsychiatric conditions and compounds other than NMDAR antagonism in MDD. This would be based on the general hypothesis that an effective drug with a large enough therapeutic window is expected to act more preferentially on disease-related connections than unrelated networks. As the NMDAR is widely expressed throughout the CNS, we did not take receptor distribution into account in the current study. However, when using this method with other CNS active compounds, the functional-anatomical distribution of the target receptor should be considered. Although more studies are required to fully understand this approach’s applicability and usefulness, it provides a promising new paradigm for applying pharmaco-MRI in drug development.

## Data Availability

The datasets presented in this article are not readily available because no restrictions apply to the dataset. Requests to access the datasets should be directed to krecourt@chdr.nl.

## Footnotes

^1^ toetsingonline.nl

^2^ http://fsl.fmrib.ox.ac.uk/fsl/fslwiki/FSLNets
