# Supplementary material for: Ketamine effects on resting state functional brain connectivity in major depressive disorder patients: a hypothesis-driven analysis based on a network model of depression
Source: Front Neurosci. 2025 Feb 3;19:1531375. doi: 10.3389/fnins.2025.1531375 (PMC11830811; doi:10.3389/fnins.2025.1531375)
Supplement: Supplementary file 1 [file Data_Sheet_1.DOCX]

Ketamine effects on resting state functional brain connectivity in major depressive disorder patients: a hypothesis-driven analysis based on a network model of depression

**Supplementary material**

**fMRI Analysis**

fMRI data were pre-processed and analyzed using Functional Magnetic Resonance Imaging of the Brain Software Library (FSL, version 5.0.9). Before pre-processing, all structural and functional images were submitted to a visual quality check. Pre-processing of the resting state images was performed using FMRI Expert Analysis Tool (FEAT). First, brain extraction^2^, motion correction using Motion Correction using FSL’s Linear Image Registration Tool (MCFLIRT)^3^, and spatial smoothing using a Gaussian kernel of FWHM 6mm were performed. Registration parameters were calculated using FLIRT^4^ and FSL’s Non-linear Image Registration Tool (FNIRT)^5,6^. To calculate the registration parameters, functional images were registered to the structural T1-weighted image using Boundary-Based Registration. The T1-weighted images were registered to the 2mm isotropic Montreal Neurological Institute (MNI)-152 standard space image using non-linear registration with a warp resolution of 10mm. Next, to further increase signal-to-noise ratio, an ICA-based strategy for Automatic Removal of Motion Artefacts (ICA-AROMA) was used correcting for secondary motion artefacts^7^. Additionally, high pass temporal filtering with a cut-of frequency of 0.01Hz was applied using FEAT. Lastly, functional images were warped to the 2mm isotropic MNI standard space image using the registration parameters calculated before.

**Regions of interest CLIPST model**

Regions of interest were defined based on prior research.^8-13^ Masks of the regions were made using either the Harvard Oxford Subcortical Atlas or by drawing spheres centred around Montreal Neurological Institute (MNI) coordinates (x, y, z, in MNI space). The following regions were defined: left and right dorsolateral prefrontal cortex (dlPFC; sphere at ± 36, 27, 29 with a 10 mm radius), left and right subgenual anterior cingulate cortex (sgACC; sphere at ± 2, 28, -5 with a 5 mm radius), left and right dorsomedial prefrontal cortex (dmPFC; sphere at left: -24, 35, 28, right: 18, 34, 29, with a 10 mm radius), left and right ventrolateral prefrontal cortex (vlPFC; sphere at ± 47, 26, 15 with a 5 mm radius), left and right ventromedial prefrontal cortex (vmPFC; sphere at left: -6, 50, -9, right: 7, 54, -9, with a 5 mm radius), left and right lateral orbitofrontal cortex (lOFC; sphere at ± 34, 42, -8 with a 5 mm radius), left and right amygdala (Harvard-Oxford probability atlas, including voxels with a probability of > 20%), left and right caudate, dorsal section (sphere at ± 13, 15, 9 with a 3.5 mm radius), left and right caudate, ventral-Nucleus Accumbens section (ventral-NAcc; sphere at ± 9, 9, -8 with a 3.5 mm radius), left and right putamen, dorsal-caudal section (sphere at ± 28, 1, 3 with a 3.5 mm radius), left and right putamen, ventral-rostral section (sphere at ± 20, 12, -3 with a 3.5 mm radius), left and right insula (sphere at left: -45, 5, 9, right: 45, 3, 15, with a 8 mm radius), left and right hippocampus (Harvard-Oxford probability atlas, including voxels with a probability of > 50%), left and right posterior cingulate cortex (PCC; sphere at ± 6, -50, 24 with a 7 mm radius).

Vago et al.^14^ describes his CLIPST model both in the form of a scheme and a supporting text. We decided that, to avoid ambiguity and make the selection of connections as straightforward as possible, to follow the following process. We took the supporting text and highlighted the places where connections were mentioned. We decided to not include the broad descriptions of connections from other models and connections with the putamen and caudate. Instead the more detailed and specific descriptions of the connections for the ventral and dorsal networks are used. These highlights are matched in the 105 unique connections grid in Supplementary Table 1. The connections were only marked as connections of interest if they were directly connected in the Vago scheme, without intermediate hubs (Figure 1). After the process described above, we checked which direct connections in the figure were not mentioned in the supporting text and added these to the grid.

**Supplementary Table 1. Connections of interest (indicated by numbers 1-39) in a 15x15 (105 unique connections) grid**

|  | dmPFC | dlPFC | omPFC | vmPFC | vlPFC | Hypothalamus | ACC | PCC | Insula | Amygdala | Hippocampus | Thalamus | Nacc | Putamen | Caudate |
| --- | --- | --- | --- | --- | --- | --- | --- | --- | --- | --- | --- | --- | --- | --- | --- |
| dmPFC | \|  \| \| --- \| | 1 | 2 | 3 | 4 |  |  | 5 |  |  |  |  |  |  |  |
| dlPFC |  |  | 6 | 7 | 8 |  | 9 |  |  |  |  |  |  |  |  |
| omPFC |  |  |  | 10 | 11 | 12 | 13 | 14 | 15 |  | 16 | 17 | 18 |  |  |
| vmPFC |  |  |  |  | 19 | 20 | 21 | 22 | 23 |  | 24 | 25 | 26 |  |  |
| vlPFC |  |  |  |  |  | 27 | 28 |  | 29 |  | 30 | 31 | 32 |  |  |
| Hypothalamus |  |  |  |  |  |  |  |  | 33 | 34 |  |  |  |  |  |
| ACC |  |  |  |  |  |  |  |  | 35 | 36 | 37 | 38 |  |  |  |
| PCC |  |  |  |  |  |  |  |  |  |  |  |  |  |  |  |
| Insula |  |  |  |  |  |  |  |  |  |  |  |  |  |  |  |
| Amygdala |  |  |  |  |  |  |  |  |  |  |  | 39 |  |  |  |
| Hippocampus |  |  |  |  |  |  |  |  |  |  |  |  |  |  |  |
| Thalamus |  |  |  |  |  |  |  |  |  |  |  |  |  |  |  |
| Nacc |  |  |  |  |  |  |  |  |  |  |  |  |  |  |  |
| Putamen |  |  |  |  |  |  |  |  |  |  |  |  |  |  |  |
| Caudate |  |  |  |  |  |  |  |  |  |  |  |  |  |  |  |

**References**

1. Jenkinson, M., Beckmann, C.F., Behrens, T.E., Woolrich, M.W. and Smith, S.M., 2012. Fsl. *Neuroimage*, *62*(2), pp.782-790.
2. Smith, S.M., 2002. Fast robust automated brain extraction. *Human brain mapping*, *17*(3), pp.143-155.
3. Jenkinson, M., Bannister, P., Brady, M. and Smith, S., 2002. Improved optimization for the robust and accurate linear registration and motion correction of brain images. *Neuroimage*, *17*(2), pp.825-841.
4. Jenkinson, M. and Smith, S., 2001. A global optimisation method for robust affine registration of brain images. *Medical image analysis*, *5*(2), pp.143-156.
5. Andersson, J.L., Jenkinson, M. and Smith, S., 2007. Non-linear optimisation FMRIB technical report TR07JA1. *Practice*.
6. Andersson, J.L., Jenkinson, M. and Smith, S., 2007. Non-linear registration, aka Spatial normalisation FMRIB technical report TR07JA2. *FMRIB Analysis Group of the University of Oxford*, *2*(1), pp.1-22.
7. Pruim, R.H., Mennes, M., van Rooij, D., Llera, A., Buitelaar, J.K. and Beckmann, C.F., 2015. ICA-AROMA: A robust ICA-based strategy for removing motion artifacts from fMRI data. *Neuroimage*, *112*, pp.267-277.
8. Dandash, O., Harrison, B.J., Adapa, R., Gaillard, R., Giorlando, F., Wood, S.J., Fletcher, P.C. and Fornito, A., 2015. Selective augmentation of striatal functional connectivity following NMDA receptor antagonism: implications for psychosis. *Neuropsychopharmacology*, *40*(3), pp.622-631.
9. Khalili-Mahani, N., Niesters, M., van Osch, M.J., Oitzl, M., Veer, I., de Rooij, M., van Gerven, J., van Buchem, M.A., Beckmann, C.F., Rombouts, S.A. and Dahan, A., 2015. Ketamine interactions with biomarkers of stress: a randomized placebo-controlled repeated measures resting-state fMRI and PCASL pilot study in healthy men. *Neuroimage*, *108*, pp.396-409.
10. McCabe, C. and Mishor, Z., 2011. Antidepressant medications reduce subcortical–cortical resting-state functional connectivity in healthy volunteers. *Neuroimage*, *57*(4), pp.1317-1323.
11. Scheidegger, M., Walter, M., Lehmann, M., Metzger, C., Grimm, S., Boeker, H., Boesiger, P., Henning, A. and Seifritz, E., 2012. Ketamine decreases resting state functional network connectivity in healthy subjects: implications for antidepressant drug action.
12. Sheline, Y.I., Price, J.L., Yan, Z. and Mintun, M.A., 2010. Resting-state functional MRI in depression unmasks increased connectivity between networks via the dorsal nexus. *Proceedings of the National Academy of Sciences*, *107*(24), pp.11020-11025.
13. Steffens, D.C., Taylor, W.D., Denny, K.L., Bergman, S.R. and Wang, L., 2011. Structural integrity of the uncinate fasciculus and resting state functional connectivity of the ventral prefrontal cortex in late life depression. *PloS one*, *6*(7), p.e22697.
14. Vago et al. CLIPST. Vago, D. R., Epstein, J., Catenaccio, E., and Stern, E. (2011). Identification of neural targets for the treatment of psychiatric disorders: the role of functional neuroimaging. *Neurosurgery Clinics* 22, 279–305.
